# Interplay of health literacy, healthcare access and health behaviors with oral health status among older persons

**DOI:** 10.3389/fpubh.2022.997987

**Published:** 2022-12-08

**Authors:** Padmore Adusei Amoah, Millicent Ofori Boateng, Adwoa Owusuaa Koduah, Princess Ruhama Acheampong

**Affiliations:** ^1^Department of Applied Psychology, School of Graduate Studies, Institute of Policy Studies, Lingnan University, Tuen Mun, Hong Kong SAR, China; ^2^Department of Community Health, Ensign Global College, Kpong, Ghana; ^3^Center of Gerontological Nursing, School of Nursing, The Hong Kong Polytechnic University, Hong Kong, Hong Kong SAR, China; ^4^Department of Health Promotion and Education, School of Public Health, Kwame Nkrumah University of Science and Technology, Kumasi, Ghana

**Keywords:** health literacy, oral health, medical check-up, access to healthcare, gender, older persons, Ghana

## Abstract

This study contributes to the ongoing debate on social determinants of oral health of older persons. Specifically, it examines the direct and indirect effects of health literacy and access to healthcare on oral health status of older persons. The study also investigates whether general health status and health behavior (routine medical check-ups) explain the association of health literacy and healthcare access with oral health status. The gender dimensions of these relationships are also explored. Data were derived from 522 participants aged 50 years and older located in five regions in Ghana. Path analyses in structural equation modeling (SEM) were used to analyse the data. General health status (β = −0.049, *p* < 0.005), medical check-up (β = 0.124, *p* < 0.01), and health literacy (β = 0.133, *p* < 0.01) were positively associated with oral health status. General health status mediated the positive relationship between health literacy and oral health status (β = 0.048, *p* < 0.01). General health status (β = 0.016, *p* < 0.05) and medical check-ups (β = 0.025, *p* < 0.05) mediated the association between access to healthcare and oral health status. The mediational role of routine medical check-up in the association between access to healthcare and oral health status was significantly stronger (B = 0.063, *p* < 0.01) among men (β = 0.051, *p* < 0.01) than women (β = 0.003, *p* > 0.05). Analyses of oral health issues among older persons in Ghana and settings alike must recognize the complex interplay among critical social determinants to initiate pragmatic health and social policy interventions.

## Introduction

Research has consistently linked poor oral health to unsatisfactory general well-being among older persons (1–3). Oral health is the “state of being free from mouth and facial pain, tooth infection and decay, tooth loss, and other diseases and disorders that limit an individual's capacity in biting, chewing, smiling, speaking, and psychosocial well-being” (4). Poor oral health predisposes people to conscious food selection to avoid uncomfortable conditions such as pain in chewing and swallowing, all of which lead to poor nutrition and ill-being (1, 5).

Many factors account for poor oral health among older persons, including biological characteristics; poverty; beliefs and practices about food and oral hygiene; and physical and cognitive abilities that affect adoption of preventive measures (2, 3, 6). Socio-demographic characteristics such as age, gender, income and educational level also affect oral health (6). However, underlying these factors are fundamental social determinants such as health literacy and access to healthcare (7–10). Access to healthcare describes factors that promote or impede the availability and utilization of preventive and curative health services (e.g., low knowledge of care services, unavailability of services, limited service options and cost of services) (11). For example, inadequate access to dental health services leads to poor oral health status (12, 13). Likewise, low health literacy—the cognitive and social skills that shape the motivation and ability of individuals to gain access to, understand, and use health information in ways that promote and maintain good health (14)—causes poor oral health (15). Older persons with sufficient health literacy adopt better self-management practices; enjoy increased knowledge of oral health issues and make timely use of needed health services (16–19).

Despite the importance of health literacy and access to healthcare to general health, evidence of how these factors affect oral health remains a subject of an ongoing investigation in low and middle-income countries (LMICs) (15–17, 20). With the majority of existing studies exploring the direct association of health literacy and access to healthcare with oral health, recent research advocates for more studies that examine the complex pathways in which these fundamental social determinants shape oral health of older persons (5, 17). For instance, oral health status is conditioned by socio-demographic factors such as gender (21, 22), but little is known about the gender dynamics in these relationships.

### This study

**Figure 1:**
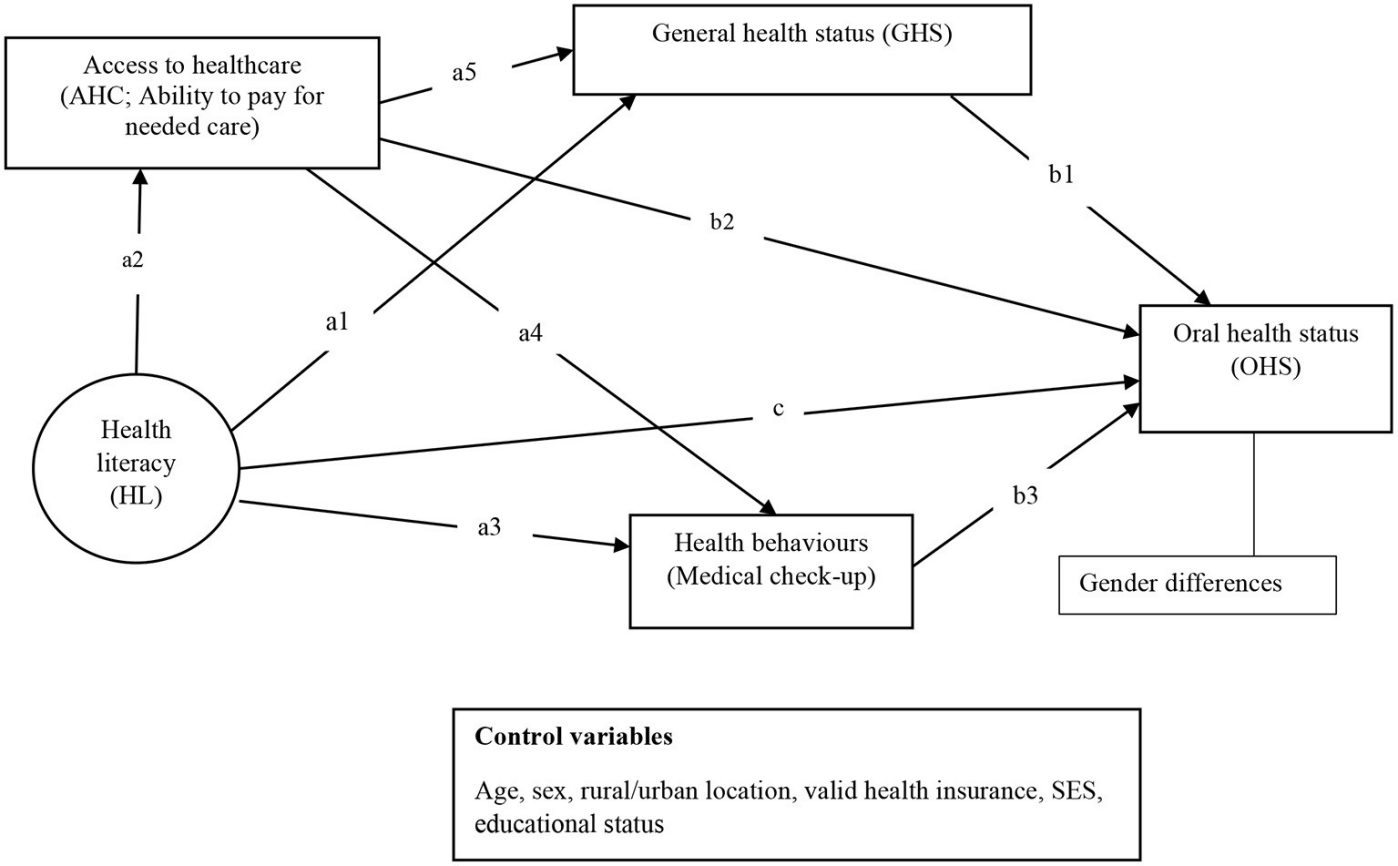
A heuristic conceptual and analytical framework underpinned by the causal pathway between health literacy and health outcomes model (11).

This study explores the interconnections among health literacy, access to healthcare, general health status and health behavior and their influence on oral health status of older persons. Specifically, as shown in Figure 1, it examines whether general health status (path a1, b1); access to healthcare (path a2, b2); medical check-ups (path a3, b3) explain the association between health literacy and oral health status among older persons in Ghana. The study also examines the extent to which general health status (path a5, b1) and health behavior (represented by medical check-ups; path a4, b3) explain the relationship between access to healthcare and oral health status. Furthermore, the study explores gender differences in the mediational relationships.

### Health literacy, access to healthcare and oral health: Theoretical and empirical perspectives

This study employs Paasche-Orlow and Wolf (11) model of the causal pathway between health literacy and health outcomes to examine the specified relationships in Figure 1. The model elucidates the systemic and interactional mechanisms by which health literacy affects health outcomes. These mechanisms include access and utilization of healthcare and people's willingness to adopt preventive healthcare practices (11). People with low health literacy interact less frequently with the health system and may lack the motivation and efficacy to adopt preventive health measures (e.g., preventive medical check-ups), all of which can lead to poor health (including poor oral health) (11, 18).

Empirical evidence shows that access to healthcare explains why and how health literacy affects health outcomes (23). This is because people with low health literacy have been less equipped to pursue self-care, resulting in poor health outcomes (11). Petersen and Yamamoto (10) have argued that affordable and appropriate oral health services must be provided to improve the well-being of older persons. While this is understandable, it raises another question, particularly in LMICs: are older persons willing, capable, and knowledgeable enough to use such services to prevent and address their oral health problems? Indeed, behaviors of older persons, including self-checked oral conditions, such as brushing their teeth and having regular dental check-ups, are likely to explain why high health literacy is associated with better oral health outcomes (17, 18). Nevertheless, the extent to which their attitudes toward medical check-ups explain the relationship between health literacy and oral health status has not been adequately examined empirically.

#### General health status and medical check-ups as mediators of access to healthcare and health literacy

The willingness of older persons to use available health services is partly predicated on the actual condition of their health and their perceptions of it (e.g., perceived severity of their health status and perception of pain) (17). Having adequate access to healthcare is associated with general health status (24).

However, because many older persons in LMICs have limited access to healthcare, their oral health likely receives the least attention compared with other medical problems (25–27). For instance, a study in Nigeria found that only 26.4% of older persons had ever visited a dentist, with most of them seeking curative care instead of preventive care (25). When other forms of physical healthcare are prioritized, oral health conditions become subordinate in the absence of pragmatic programmes to promote oral health (27). Neglect of oral health has negative consequences for overall physical health. Thus, general health status is likely to explain the influence of access to healthcare on oral health status, considering that oral health conditions often precede or trigger other health conditions (28). Moreover, the influence of access to healthcare on oral health can depend on the kind of treatments sought (e.g., preventive or curative care) (25). However, it is not well-investigated as regards the bearing that choices about preventive vs. curative care have on oral health outcomes. Additionally, much is not known about the mechanism that connects access to healthcare to oral health status among older persons in LMICs.

Furthermore, while health literacy and access to healthcare are critical to the oral health of older persons, gender dynamics also affect the association of health literacy and access to health care with oral health of older persons. However, this aspect is not included in the Paasche-Orlow and Wolf (11) causal model on health literacy and health. Studies show contrasting perspectives, with some indicating a high prevalence of poor oral health status among older men than women (21, 29) and vice versa (6). Sociocultural norms and practices affect the choices men and women make relative to their health and well-being (30, 31). For instance, women tend to have low health literacy in some low- and middle-income countries due to prolonged inadequate formal education for females (32). Inadequate health literacy can reduce access to needed health services and peoples' ability to engage in preventive healthcare (22, 30, 33).

Notwithstanding, ongoing social changes are transforming the conventional roles of men and women even in highly patriarchal societies, which have implications for health and health-related decisions (34). Indeed, research on oral health is increasingly finding fewer differences between older men and women (29). Such differences indicate a need for context-specific studies to learn more about gender disparities and other social determinants of older persons' oral health outcomes.

### Hypotheses

Based on the discussions above, this study has drawn the following hypotheses:

- H1: Health literacy (path c) and access to healthcare (path b2) will be positively associated with oral health status.
- H2: General health status (path a1, b1), access to healthcare (path a2, b2), and medical check-ups (path a3, b3) will mediate the relationship between health literacy and oral health status.
- H3: General health status (path a5, b1) and medical check-up (path a4, b3) will mediate the relationship between access to healthcare and oral health status.
- H4: The specified mediational relationships (H2 and H3) are likely to be stronger among men than women.

## Methodology

This study is based on data generated as part of broader research on the social aspects of health and well-being among the general population in Ghana. The research involved a cross-sectional survey carried out across five of the then 10 administrative regions of Ghana from July to August 2018. The study adopted a multi-stage cluster sampling approach to derive participating regions, districts, communities, and respondents. A purposive sampling technique was used to select the regions, districts and communities involved. The criteria for selection included geographical diversity (e.g., considering regions from southern, middle and northern parts of the country as well as rural and urban locations); low, medium and high socioeconomic groups; religious characteristics and ethnic characterizes of the country to achieve a balanced sample. With these characteristics in mind, the study selected 29 districts across the five regions as follows: Brong Ahafo (five districts), Greater Accra (seven districts), Eastern (three districts), Ashanti (eight districts), and Northern (six districts) regions.

### Data collection

There were 128 communities involved in the study, including 51 and 77 from rural and urban areas, respectively. Participants were sampled from households using a systematic sampling technique. Only participants who had lived in their communities for at least 12 months preceding the study were included in the survey. Based on previous experiences, the study selected one person from every second and fifth house in rural and urban areas, respectively, to participate in the survey. This systematic criterion was primarily born out of previous studies (35, 36), which indicated that residents in the same houses often share similar socioeconomic characteristics and sometimes familial relations. The sampling process yielded 2,097 respondents aged 15 years and older. Detailed information about the sample size computation has been reported elsewhere (15) and attached as a flow chart in Appendix I. Trained interviewers assisted in collecting data from householders who consented to participate in the survey. This present study analyses the responses of 522 older persons from the broader research. Older persons in this study refer to people aged 50 years and above because the study was conducted in a country where life expectancy is relatively low (15, 36, 37). The Research Ethics Committee of Lingnan University approved the study protocol (EC-043/1718). All participants provided either written or verbal consent before enrolling on the study.

### Measures

#### Dependent variable

Oral health status was measured by a single item. Respondents were asked, “How would you rate your oral health in the past 12 months?” They responded on a five-point Likert scale ranging from 1 = very poor to 5 = very good. The WHO (4) definition of oral health was appended to the question for respondents' easy reference. Although this is a single-item scale and also based on self-report, the approach has been used severally to measure oral health status of different population groups. It has shown consistent results with clinical diagnoses (38).

#### Independent variables

*Health literacy:* This study used the Swedish Functional Health Literacy scale (SFHL), which focuses on a person's ability to assess, read, understand, and apply health information. It comprises five items measured on a five-point Likert scale: “always,” “often,” “sometimes,” “not often,” and “never.” The items include: “Do you ever ask someone else to read or explain health information? Do you think that it is difficult to read health information because the text is difficult to see (even if you have glasses or contact lenses)?”; and “Do you think that it is difficult to understand words or numbers in health information?” (39, 40). To make the instrument contextually relevant, it was adapted through back-translation (English–Twi–English) and pretested. In the process, minor changes were made. For example, the response option “seldom” was changed to “not often” and “all the time” replaced “always” in the original scale, as these were easier for respondents to understand (41). This instrument showed a high level of reliability with a Cronbach alpha of 0.92 in this study. In the descriptive analyses, the responses were summed to reach a maximum score of 25 and a minimum of 5. However, the full scale was used as a latent variable in a subsequent structural equation model. A confirmatory factor analyses showed that the instrument has a high construct validity and theoretical fit considering these indices [see (42)]: Minimum discrepancy function by degrees of freedom divided (CMIN/df)= 1.525 (*p*-value = 0.206), goodness-of-fit index (GFI) = 0.996, adjusted goodness-of-fit index (AGFI) = 0.980, incremental fit index (IFI) = 0.999, root mean square error of approximation (RMSEA) = 0.034, and comparative fit index (CFI) = 0.999.

*Access to healthcare*, which was also used as a mediator, was measured using a one-item instrument. The item focused on economic access to healthcare. Participants were asked this question: “During the past 12 months, did it ever happen that you did not get the medical treatment you needed because you could not pay for it?” They answered on a four point-Likert scale: (1) “Never,” (2) “not often,” (3) “often,” and (4) “all the time.” This item was taken from a well-tested instrument on access to healthcare (43, 44). Response to “never” indicated high access to healthcare. The question focused on the economic aspect of healthcare because it is a fundamental determinant of access to healthcare, especially among socioeconomically disadvantaged groups such as older persons (12). Even in high-income societies such as the US, oral health status is considered a symbol of social inequality across the life course due to the significant impact of socioeconomic status on oral healthcare (45). Poor oral health is common among older persons who cannot afford needed treatment (12).

#### Mediators

*General health status:* The participants were asked to rate their general health status (including their physical and mental health) in the past 4 weeks. They rated their health on a five-point Likert scale; “poor,” “fair,” “good,” “very good,” or “excellent.” Longitudinal studies show that this measure can predict mortality in old age (46).

*Medical check-up behaviors*: Participants were asked to indicate if they had visited a doctor for a routine medical check-up in the past 24 months? A routine medical check-up was defined as a general physical exam, not an exam for a particular injury, illness, or condition, and not by a doctor's recommendation. They answered either “yes” or “no.” This question was adapted from the East Asian Social Survey (47). However, it is worth noting that some participants included unofficial consultations they had held with health practitioners and voluntary consultations for previously diagnosed chronic conditions.

#### Covariates

These variables were included as covariates as derived from the Paasche-Orlow and Wolf (11) model of the causal pathway between health literacy and health outcomes: age (measured in years), sex (male or female), educational attainment, which was treated as an ordinal variable (never been to school, primary school, middle school, secondary school, tertiary), location of residence (rural or urban), and whether a person had health insurance (in reference to Ghana's National Health Insurance Scheme, NHIS) (48). The study also controlled for the monthly income/stipend (in Ghana Cedis) and respondents' socioeconomic status using the MacArthur one-item scale (49). This scale required respondents to rate their general social and economic conditions compared to others in their social circles from 1 = low to 10 = high.

### Data analyses

The data analyses comprised a descriptive analysis that provided an overview of the characteristics of the measured variables (Table 1). This was followed by a Spearman's Rank correlation analysis (Table 2) to select covariates for the inferential analyses (i.e. socio-demographic variables associated with oral health status). The inferential analyses employed a structural equation modeling (SEM) technique using SPSS AMOS version 26 to test the specified model (Figure 1). Before the SEM, all missing responses (around 2% of responses across variables) were replaced by the mean of the variables concerned. All variables were standardized before including them in the SEM to help reduce potential multicollinearity. A confirmatory factor analysis was conducted to test for the model fit for the SFHL and the general analytical model (Figure 1). The model fit was evaluated following the cut-off suggested by Byrne (42) for CMIN/df, GFI, AGFI, IFI, RMSEA, and CFI.

**Table 1:** Descriptive statistics of variables in the study.

**Variable**	**Men**		**Women**			**General**	
	**Mean/Valid *n* (n = 275)**	**SD/%**	**Mean/Valid *n* (*n* = 247)**	**SD/%**	***p*-value**	**Mean/Valid *n* (*n* = 522)**	**SD/%**
**Age (in years)**	61.5	8.3	61.0	9.3	0.525^b^	61.28	8.81
*Minimum-maximum*	50–85		50–91			50–91	
**Location of residence**					0.910		
Rural	120	43.6	109	44.1		229	43.9
Urban	155	56.4	138	55.9		293	56.1
**Socioeconomic status (SES)**	4.44	1.7	4.1	1.6	**0.030^**b**^**	4.3	1.7
*Minimum-maximum*	1–10		1–9			1–10	
**Region of residence**					0.108		
Ashanti	78	28.4%	63	25.5%		141	26.2
Greater Accra	31	11.3%	34	13.8%		65	18.0
Brong Ahafo	52	18.9%	50	20.2%		102	16.1
Northern Region	71	25.8%	45	18.2%		116	20.9
Eastern Region	43	15.6%	55	22.3%		98	18.8
**Educational attainment**					**0.001**		
Never been to school	81	29.5	106	42.91		187	35.8
Primary school	57	20.7	62	25.1		119	22.8
Middle School	71	25.8	42	17.0		113	21.6
Secondary School	38	13.8	24	9.7		62	11.9
Tertiary	28	10.1	13	5.3		41	7.9
Monthly Income (GH 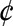 )*^a^*	436.74	864.42	291.20	369.89	**0.014** ^**L**^	363.97	617.16
*Minimum-Maximum score*	15–5,500		7–4,000			7–5,500	
**Health insurance**					**0.027**		
No	116	42.2	81	32.8		197	37.7
Yes	159	57.8	166	67.2		325	62.3
**Health literacy (HL)**					**0.000** ^**b**^		
*Mean (SD)*	18.3	4.6	16.1	5.8		17.1	5.3
*Minimum-Maximum score*	*5–25*		*5–25*			*5–25*	
**Medical check-up**					0.858		
No	194	70.5	176	71.3		370	70.9
Yes	81	29.5	71	28.7		152	29.1
**Access to healthcare**					**0.431** ^**b**^		
All the time	7	2.5	10	4.0		17	3.3
Often	60	21.8	63	25.5		123	23.6
Not often	54	19.6	35	14.2		89	17.0
Never	154	56.0	139	56.3		293	56.1
**General health status**					**0.000** ^**b**^		
Poor	27	9.8	43	17.4		70	13.4
Fair	107	38.9	101	40.9		208	39.8
Good	88	32.0	81	32.8		169	32.4
Very good	40	14.5	16	6.5		56	10.7
Excellent	13	4.7	6	2.4		19	3.6
**Oral health status**					**0.016^**b**^**		
Very poor	8	2.9	10	4.0		18	3.5
Poor	33	12.0	42	17.0		75	14.3
Neither poor nor good	60	21.8	75	30.4		135	25.8
Good	125	45.5	92	37.2		217	41.6
Very good	49	17.8	28	11.3		77	14.8

a GH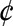 1 = US$ 0.183; Bold figures: *p*-values < 0.05;

b *p*-value based on independent sample t-test.

L *p*-value is based on the likelihood ratio due to the relatively low response rate. All other *p*-values are based on Pearson Chi-Square tests.

**Table 2:** Spearman's correlation analyses of variables in the study.

		**1**.	**2**.	**3**.	**4**.	**5**.	**6**.	**7**.	**8**.	**9**.	**10**.	**11**.	**12**.	**13**.
		**Oral health status**	**Health literacy**	**Access to healthcare**	**Medical check-up**	**General health status**	**Region of residence**	**Educational attainment**	**SES**	**Health insurance**	**Sex (Male)**	**Area of residence (Rural)**	**Income**	**Age**
1.	Oral health status	1.000												
2.	Health literacy	−0.218**	1.000											
3.	Access to healthcare	−0.002	0.103*	1.000										
4.	Medical check-up	0.133**	−0.055	0.210**	1.000									
5.	General health status	0.283**	−0.279**	−0.103*	0.082	1.000								
6.	Region of residence	0.090	−0.189**	−0.173**	−0.101*	0.087	1.000							
7.	Educational attainment	0.057	−0.169**	0.047	0.064	0.131**	−0.410**	1.000						
8.	SES	0.113*	−0.094*	0.024	0.043	0.148**	−0.342**	0.605**	1.000					
9.	Health insurance	−0.093*	−0.048	−0.095	0.257**	−0.021	−0.084	0.070	−0.025	1.000				
10.	Sex (Male)	0.187**	−0.185**	0.012	0.009	0.159**	0.213**	0.075	0.051	−0.028	1.000			
11.	Area of residence (Rural)	−0.041	−0.042	0.130**	−0.083	−0.169**	−0.017	−0.108*	−0.069	0.100*	−0.033	1.000		
12.	Income	0.022	−0.005	−0.150*	0.145*	0.104	−0.404**	0.270**	0.339**	0.068	−0.145*	−0.071	1.000	
13.	Age	−0.092*	0.052	−0.104*	0.026	−0.280**	0.060	−0.237**	−0.131**	−0.018	0.023	0.113*	−0.154*	1.000

** Correlation is significant at the 0.01 level (2-tailed).

* Correlation is significant at the 0.05 level (2-tailed).

Following the results of the mediational analyses specified in Figure 1, the analytical tools offered by Gaskin (50) were used to conduct a moderated mediation analysis which tested for the differences between men and women for all significant mediational paths observed. All significant associations in the study were evaluated at *p* < 0.05.

## Results

The study included respondents aged 61 years of age on average, with no difference between men and women regarding their age. They were located in both rural and urban areas. The participants predominantly had primary and middle school education as their highest educational attainments. The men had higher educational attainments than women. More women (67.2%) had valid health insurance than men (57.8%). The participants' health literacy was low to moderate, with most of them scoring an average of 17.1 out of 25. Men had higher health literacy than women. About 29.1% of respondents had undertaken a medical check-up in the 24 months before the study with no difference in gender. About 26.9% of them had had challenges in accessing healthcare. Approximately 53.2% of respondents described their general health negatively, while about 43.6% reported their oral health status as very poor, poor or neither poor nor good. Men reported better general status and oral health status than women as shown in Table 1. According to Table 2, only age, sex, having valid health insurance and socioeconomic status were significantly correlated with the oral health status.

**Figure 2:**
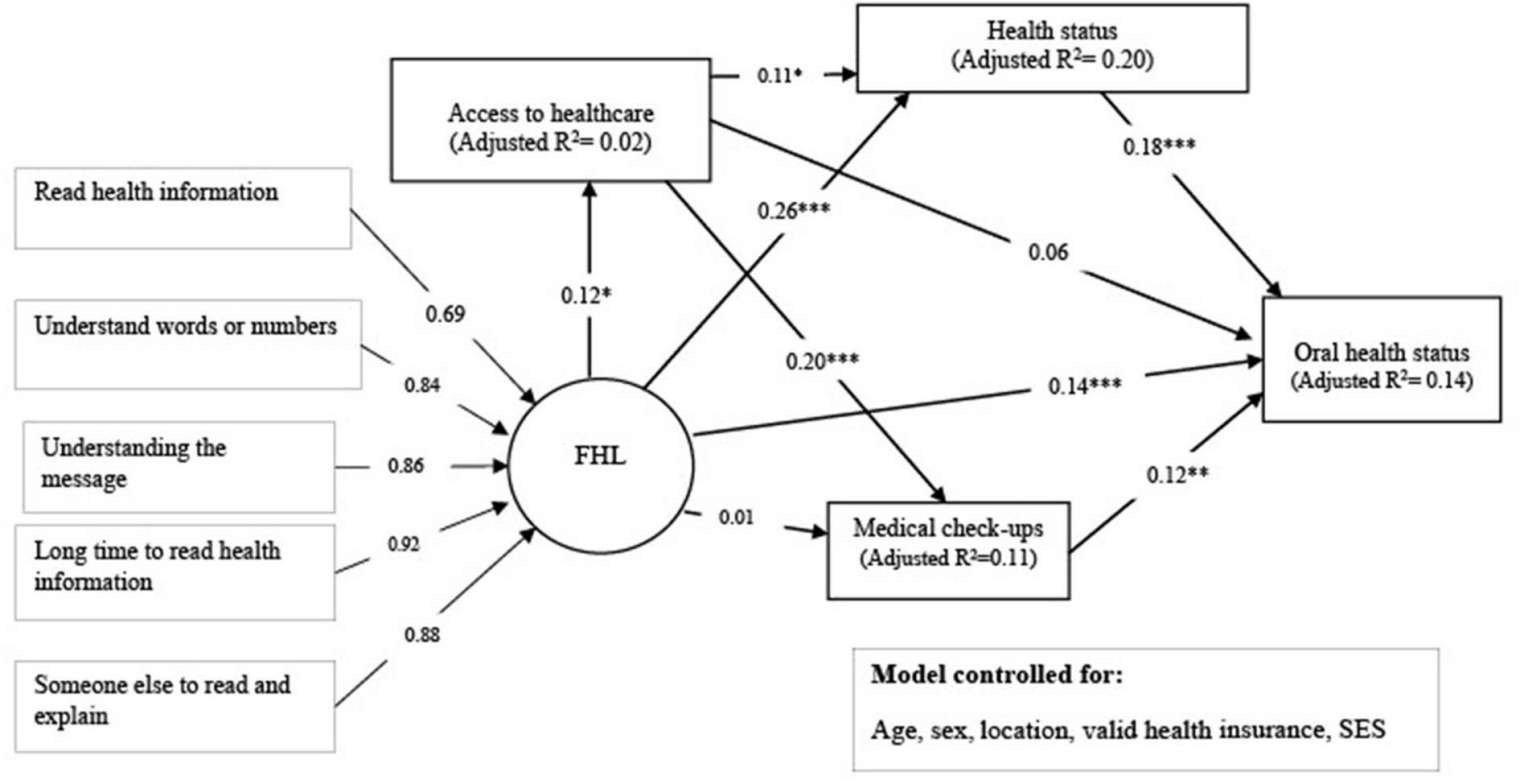
The association of health literacy, general health status, access to healthcare, and medical check-ups with oral health literacy by SEM. Coefficients are based on standardized estimates. ***p < 0.001; **p < 0.01; *p < 0.05. Model fit indices: CMIN/df = 1.920 (*p*-value = 0.000), GFI = 0.976, AGFI = 0.939, CFI = 0.982, IFI = 0.982, RMSEA = 0.045.

From Table 3 (see also Table 4), general health status (β = −0.049, *p* < 0.005), medical check-up (β = 0.124, *p* < 0.01), and health literacy (β = 0.133, *p* < 0.01) were associated with oral health status. The various associations between the primary variables in the study are also shown in Figure 2.

**Table 3:** Association of health literacy, medical check-up, general health status, and access to healthcare with oral health status (derived from the SEM).

**Variable**	**B**	**β**	**Std. Error**	***p*-value**
Age	−0.006	−0.049	0.005	**0.003**
**Sex**				
Males	0.226	0.111	0.091	**0.013**
Female (ref)				
**Health insurance**				
Yes	0.307	0.113	0.123	**0.012**
No (ref)				
SES	0.028	0.055	0.023	0.218
General health status	0.197	0.184	0.052	**0.000**
Medical Check–up	0.260	0.124	0.097	**0.007**
Access to healthcare	0.076	0.065	0.053	0.157
Health literacy	0.133	0.144	0.045	**0.003**
*Adjusted R-Square*		*0.138*	

Bold figures indicate significant association.

**Table 4:** Direct, indirect, and total effects of mediation paths examined in this study.

	**Health literacy**		**Access to healthcare**		**Health status**		**Medical check-up**	
	**Total effect**	**Direct effect**	**Total effect**	**Direct effect**	**Total effect**	**Direct effect**	**Total effect**	**Direct effect**
Access to healthcare	0.125**	0.125**	–	–	–	–	–	–
Health status	0.247**	0.258**	0.086*	0.086*	–	–	–	–
Body check-up	0.040	0.015	0.199**	0.199**	–	–	–	–
Oral health status	0.186**	0.144**	0.056	0.065	0.184**	0.184**	0.124*	0.124*

** *p* < 0.01;

* *p* < 0.05. All values are standardized estimates.

Tables 4, 5 show the total, direct and indirect associations between the independent variables, mediators, and oral health status. It was found that general health status mediated the positive relationship between health literacy and oral health status (β = 0.048, *p* < 0.01). General health status (β = 0.016, *p* < 0.05) and medical check-ups (β = 0.025, *p* < 0.05) mediated the association between access to healthcare and oral health status, as depicted in Table 5. Further analysis, as shown in Table 6, found that the mediational role of medical check-up behaviors in the association between access to healthcare and oral health status was significantly stronger (B = 0.063, *P* < 0.01) among males (β = 0.051, *p* < 0.01) than females (β = 0.003, *p* > 0.05). The other mediational observations did not differ by gender, according to Table 6.

**Table 5:** Indirect effects of health literacy and access to healthcare on oral health status of older persons.

**Paths**	**B**	**95% Confidence interval**		**β**	***p*-value**
		**Lower boundary**	**Upper boundary**		
Health literacy 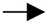 general health status 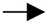 oral health status	0.044	0.023	0.071	0.048	**0.001**
Health literacy 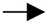 access to healthcare 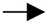 oral health status	−0.007	−0.021	0.000	−0.008	0.089
Health literacy 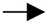 medical check-up 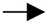 oral health status	0.002	−0.006	0.013	0.002	0.626
Access to healthcare 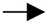 general health status 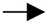 oral health status	0.019	0.005	0.039	0.016	**0.019**
Access to healthcare 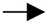 medical check-ups 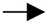 oral health status	0.029	0.009	0.057	0.025	**0.010**
Model r-square	0.14				

Bold figures indicate significant relationships. Table generated through software provided by Gaskin and Lim (51).

**Table 6:** Gender differences in the observed indirect effects of access to healthcare and oral health status of older persons.

**Paths**	**Men**			**Women**			**Gender difference** ^**∧**^	
	**B (95% Confidence interval)**	**β**	***p*-value**	**B (95% Confidence interval)**	**β**	***p*-value**	**B (95% Confidence interval)**	***p*-value**
Health literacy 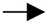 General health status 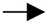 oral health status	0.069 (0.031, 0.115)	0.087	**0.002**	0.022 (0.072, 0.110)	0.018	0.110	0.040 (-0.010, 0.089)	0.185
Access to healthcare 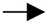 General health status 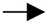 oral health status	0.015 (0.042, −0.004)	0.013	0.187	0.033 (0.005, 0.087)	0.027	**0.036**	0.014 (−0.021, 0.062)	0.530
Access to healthcare 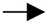 Medical check-up 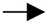 oral health status	0.057 (0.023, 0.105)	0.051	**0.004**	0.004 (−0.029, 0.017)	0.003	0.644	0.063 (0.021, 0.114)	**0.009**

Bold figures indicate significant relationships. ^∧^The gender difference was computed using the resource offered by Gaskin (50).

## Discussion

Oral health is considered critical to older persons' health and well-being. However, a dearth of knowledge on its determinants among older persons in LMICs means that older persons in these places remain vulnerable to complications around poor oral health. This study has examined the associations and pathways in which health literacy and access to healthcare affect the oral health of older persons through general health status and routine medical check-ups.

Consistent with hypothesis I, general health status, medical check-ups, and health literacy were positively associated with oral health status. According to the Paasche-Orlow and Wolf (11) health literacy causal model, having sufficient health literacy improves knowledge about existing services and health system operations, which leads to better [oral] health status. This finding is consistent with observations of other studies that place health literacy as central to the oral health of older persons in China (19) and the US (20). The association between routine medical check-up behavior with oral health status supports claims in the causal model of health literacy and health outcomes that individual characteristics (e.g., self-efficacy in managing one's health) dictate health outcomes (i.e., oral health in this case) (11). This finding implies that promoting uptake of preventive care is likely to yield positive results for oral health.

Consequently, unaddressed barriers to accessing such services must be addressed. While specialist care for oral problems is often scarce in LMICs (4, 52), older persons must at least be served with adequate information on available services and relevant social policy interventions (e.g., financial support) to motivate them to proactively care for their oral health (13).

### General health status positively mediates health literacy and oral health

While extant studies have established a strong relationship between health literacy and oral health outcomes (15, 17), this finding, which partly confirms hypothesis II, adds an important explanation to this relationship: the mediating role of general health status. Among older persons in Ghana, health literacy is likely to be critical to the oral health of those who possess or at least perceive positive overall health status. One could argue that the application of health literacy skills to improve oral health may happen as part of an attempt to address existing health conditions or maintain good health. Thus, even among older persons with sufficient health literacy, oral health is probably not a priority condition since health literacy leads to overall positive health outcomes of which oral health can be a part. This explanation is consistent with views from other studies that have argued about the limited priority of oral health among older persons (25–27). This finding means that health promotion about older person's oral health that centers around health literacy must target specific aspects of health literacy (e.g., oral health literacy) to have a more assured outcome (17). Studies show that targeted interventions, including ‘teaching and learning opportunities for oral health', are more likely to produce positive outcomes [(27), p. 9].

Nevertheless, oral health complications must be addressed with significant consideration for other health problems of older persons. Otherwise, well-intended oral health programmes will likely yield minimal impact even among those with sufficient health literacy. To this end, integrated oral healthcare programmes are critical to achieving positive oral health outcomes, especially among potentially vulnerable groups such as older persons (27). Such integrated programmes can consider oral care, screening, oral health literacy, and perhaps most importantly, the overall health conditions of older persons (27). The integrated approach can ensure that health conditions that could cause periodontal diseases, such as diabetes and cardiovascular diseases, can be managed with cognisance of the potential consequences of oral health conditions and vice versa (28).

### General health status and medical check-ups mediate association between access to healthcare and oral health status

Similar to its role in the relationship between health literacy and oral health, general health status mediated the association between access to healthcare and oral health status as predicted by hypothesis III. Considering the high priority given to general health status instead of oral health, older persons are likely to access healthcare for physical health conditions other than oral health. This is partly due to poor access to health services among this group, particularly in areas of affordability and availability of such specialized care (27, 28). With difficulties in accessing oral healthcare (52), the use of dental care becomes reactive (i.e., when there is a problem) instead of preventative action for many older persons (27). From this perspective, the influence of healthcare access on health care might depend on the extent of utilization of services for other health problems.

Nevertheless, this study's evidence also shows that having greater access to healthcare enables older persons to submit to medical check-ups that positively affect oral health. As this study did not precisely measure use of oral health check-ups, one could assume that oral health is one of the issues that may receive attention during a routine medical check-up. Additionally, given the role of general health status in the association between access to healthcare and oral health, it is also possible that oral health check-ups may be an appendage to the use of other health services. Nevertheless, these findings indicate that expanding access to general health services is a meaningful way to promote preventative oral health care behaviors. Even without dedicated oral health services, expanding healthcare access can create opportunities for older persons at risk of oral health problems to avoid deterioration of their conditions.

### Gender and the relations between access to healthcare and oral health status

Although not all the significant mediations observed differed by gender, the findings support hypothesis IV that the mediational role of medical check-ups was stronger among men than women. In a place where men are likely to be more educated and often more economically advantaged than women (53), it is not entirely surprising that medical check-ups had a significant impact on men than women regarding oral health. Although there was no difference between men and women in this study concerning general routine medical check-ups, it is possible that men used more oral health services as opposed to women, whose use of preventive oral healthcare may occur by chance—probably, as part of consultations for other health services. Even evidence in high-income countries suggests that older women struggle to access oral healthcare and afford dental insurance compared to older men (28).

Moreover, considering that men often have better health literacy than women, as reported in this study and elsewhere (32), men are likely to be more knowledgeable and proactive about seeking preventive oral care. Older persons sometimes believe that dental health check-ups and even treatments are less relevant for the aged (13). This shows a gap in oral health literacy, which might explain the difference between men and women in this instance. Nevertheless, other studies show that having comprehensive knowledge of oral health is not associated with use of dental health services (54). Hence, future studies should explore the nuances of how men and women adopt preventive care services, specifically oral health care, relative to their health literacy and the extent of their access to healthcare to provide a platform for an informed decision.

## Conclusion

Oral health is critical for the overall health and well-being of older persons. However, it is affected by many social factors that must be understood in the context of a given health and social service system to offer practical support. This study has examined the mechanism connecting two fundamental social determinants of oral health among older persons, health literacy and access to healthcare. Health literacy was associated with oral health status through general health status. General health status and routine medical check-ups mediated the relationship between access to healthcare and oral health status. Furthermore, the mediation of routine medical check-ups in the association between access to healthcare and oral health status was stronger among men than women.

These findings imply that the promotion of oral health of older persons must involve an awareness of the individual and, as much as applicable, social characteristics that shape the ability and willingness of older persons to use planned services. Given these findings, and consistent with the Paasche-Orlow and Wolf (11) causal model of health literacy, oral health promotion must be linked to individual health-related self-management skills, knowledge of oral health problems, the barriers to accessing preventive and curative services as well as older persons' overall health conditions. Moreover, the gender dimension of oral health status must be considered in oral health promotion, particularly concerning preventive oral healthcare use. This is because attitudes toward preventive health services can be pivotal in explaining how oral healthcare patterns determine overall well-being among men and women. Altogether, these findings imply that an analysis of oral health issues among older persons in Ghana and similar settings must recognize the complex interplay among critical social determinants in the places of interest to understand better and initiate practicable interventions.

### Limitations of the study

This study has provided a deeper understanding of the state and characteristics of oral health among older persons in Ghana. The findings are also relevant to older persons in similar settings in sub-Saharan Africa. While the results are instructive for public and social policy measures, it is important to be mindful of their limitations. First, this study is based on cross-sectional data. Therefore, causal inferences cannot be drawn from the findings, although they are mostly consistent with existing studies. Second, all the variables in the study were self-reported. Because of this, it is possible that the data may not entirely reflect the situation of participants due to inconsistencies in responses. Notwithstanding, extant evidence shows that self-reported data can be effective in understanding the condition of people in health-related studies [e.g., (38)]. Third, variables such as health literacy, access to healthcare, and routine medical check-ups were measured generically rather than using instruments that focus specifically on oral health. This raises a question about the direct relevance of these variables to oral health promotion strategies. However, the general measurement of the variable does demonstrate the robustness of the association of health literacy, access to healthcare and regular medical check-ups with oral health and the need to pay more attention to them in formulating health promotion strategies.

## Data availability statement

Requests for the raw data supporting the conclusions of this article will be considered on a case-by-case basis. Requests to access the datasets should be directed to PAA (pamoah@LN.edu.hk).

## Ethics statement

The Research Ethics Committee of Lingnan University, Hong Kong, approved the study protocol (EC-043/1718). The participants provided their written or oral informed consent to participate in this study.

## Author contributions

PAA conceived this study and led in preparing the manuscript, including data analyses and discussions. MOB provided support in data presentation and discussion. AOK helped in data analyses and conceptual framework of the study. PRA supported in drafting the introduction and discussion of the article. All authors contributed to the article and approved the submitted version.

## Funding

This work was supported by the Lingnan University Faculty Grant [102159].

## Conflict of interest

The authors declare that the research was conducted in the absence of any commercial or financial relationships that could be construed as a potential conflict of interest.

## Publisher's note

All claims expressed in this article are solely those of the authors and do not necessarily represent those of their affiliated organizations, or those of the publisher, the editors and the reviewers. Any product that may be evaluated in this article, or claim that may be made by its manufacturer, is not guaranteed or endorsed by the publisher.
