# Supplementary material for: Interplay of health literacy, healthcare access and health behaviors with oral health status among older persons
Source: Front Public Health. 2022 Dec 8;10:997987. doi: 10.3389/fpubh.2022.997987 (PMC9784911; doi:10.3389/fpubh.2022.997987)
Supplement: Supplementary file 1 [file Presentation_1.pdf]

Appendix I

Interplay of health literacy, healthcare access and health behaviours with oral health status among older persons

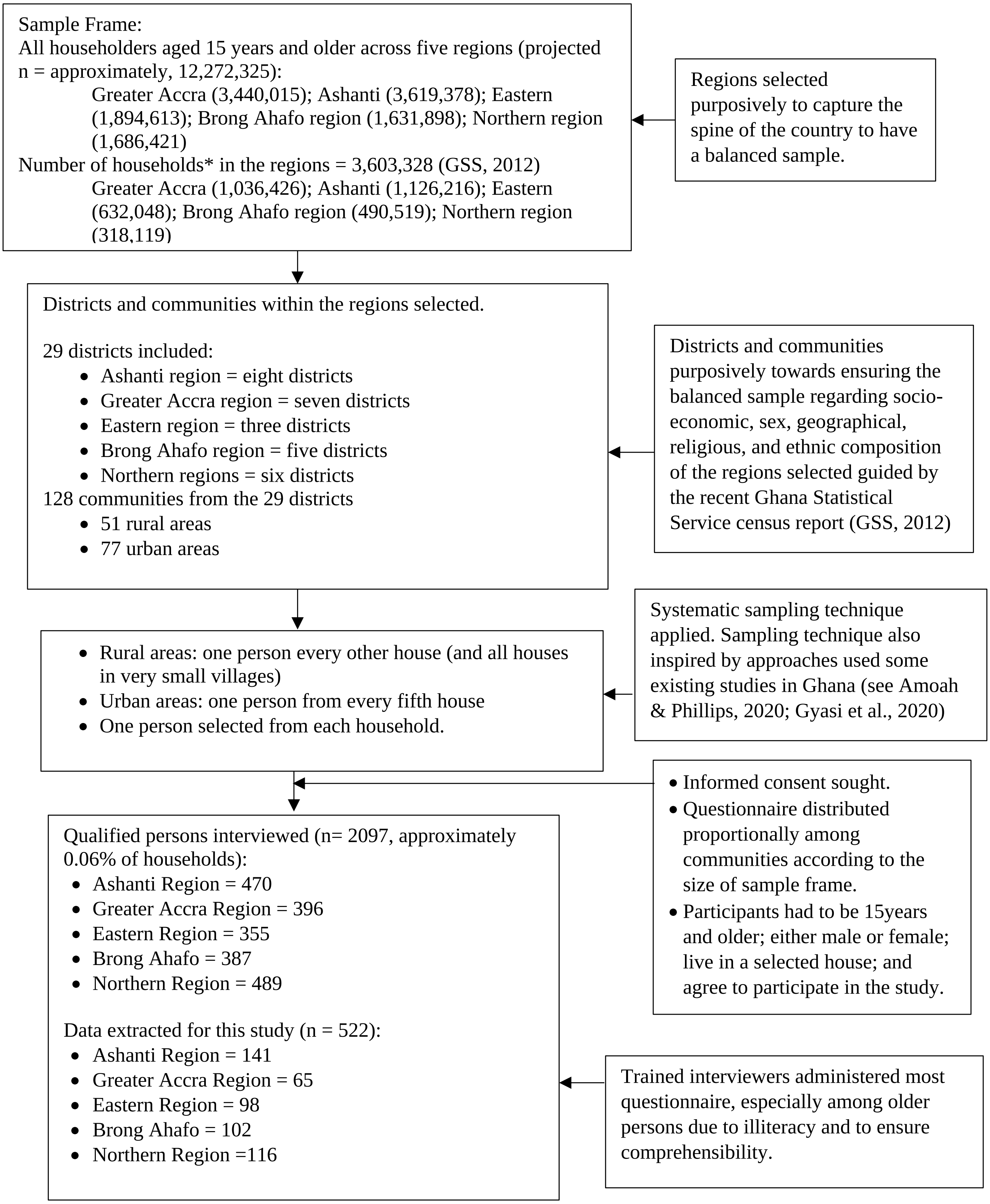

Figure 1: Flow chart of the Sampling Process of the Study

*Note: Household was defined in accordance with the Ghana Statistical Service's as: "A household was defined as a person or a group of persons, who lived together in the same house or compound and shared the same house-keeping arrangements. In general, a household consisted of a man, his wife, children and some other relatives or a house help who may be living with them. However, it is important to remember that members of a household are not necessarily related (by blood or marriage) because non-relatives (e.g. house helps) may form part of a household"(GSS, 2012, p. x).

Reference

Amoah, P. A., & Phillips, D. R. (2020). Socio-Demographic and Behavioral Correlates of Health Literacy: A Gender Perspective in Ghana. *Women & Health*, *60*(2), 123-139. doi: 10.1080/03630242.2019.1613471

GSS. (2012). 2010 Population and Housing Census: Summary report of final results. Accra: Ghana Statistical Service (GSS).

Gyasi, R. M., Phillips, D. R., & Amoah, P. A. (2020). Multidimensional Social Support and Health Services Utilization Among Noninstitutionalized Older Persons in Ghana. *Journal of Aging and Health*, 0898264318816217. doi: 10.1177/0898264318816217
